# Assessing nurses’ knowledge and attitudes towards promoting female condom use in South African primary healthcare clinics

**DOI:** 10.1186/s12913-023-10504-9

**Published:** 2024-01-05

**Authors:** Enwongo Ettang, Parimalaranie Yogeswaran, Oladele Vincent Adeniyi

**Affiliations:** Department of Family Medicine and Rural Health, Faculty of Medicine & Health Sciences, Walter Sisulu University, Mthatha, Eastern Cape South Africa

**Keywords:** Barrier method, Contraceptive knowledge, Female condoms, Nurses, STI/HIV prevention

## Abstract

**Background:**

Female condoms protect against unplanned pregnancies and sexually transmitted infections (STIs) including HIV; however, their uptake is very low in South Africa. Nurses are frontline healthcare workers and are uniquely positioned to promote their use to their clients. This study assesses nurses’ knowledge of, attitudes to, and practices regarding the promotion of female condoms at selected primary healthcare facilities in the King Sabata Dalindyebo sub-district of the Eastern Cape, South Africa.

**Methods:**

A descriptive cross-sectional study was conducted from April to May 2021 at five community health centres in the King Sabata Dalindyebo sub-district, South Africa. A total of 139 nurses completed a self-administered questionnaire. Data were analysed using simple descriptive statistics.

**Results:**

The majority of the participants (82.7%) were knowledgeable about the female condom. Some participants did not have a good attitude and willingness to promote female condom use to their clients. Junior nurses (enrolled nursing assistants and newly qualified professional nurses) were less knowledgeable about the female condom than more qualified and older nurses. There was no significant association between level of knowledge and attitude or willingness to promote the use of the female condom.

**Conclusion:**

This study found good knowledge of the female condom among the nurses; however, the knowledge did not translate into a willingness to promote the device at their health facilities. Capacity building of the junior nurses will fill the knowledge gaps identified. Studies exploring the sociocultural issues around the female condom are needed in the region.

**Supplementary Information:**

The online version contains supplementary material available at 10.1186/s12913-023-10504-9.

## Introduction

South Africa has the highest prevalence of HIV globally, with women disproportionately affected [1]. There is also a body of evidence showing an increasing trend of unplanned pregnancies in both rural and urban communities of South Africa [2]. The double burden of HIV infection and unplanned pregnancies is inherently linked to unprotected sexual intercourse between partners [3]. There are limited biomedical strategies that can simultaneously prevent HIV, other sexually transmitted infections (STIs) and unplanned pregnancies. Evidence suggests that condoms offer double protection against STIs, including HIV, and unplanned pregnancies [4]. However, available data indicates that this effective strategy is an underutilised resource in the country’s prevention efforts, especially in rural communities [5].

Worse still, the female condom, a self-initiated barrier method for pregnancy and STI prevention, is disproportionately underutilised in relation to the male condom [6]. Plausible theories for the differential rates of use could be lack of awareness of the female condom’s efficacy, inconvenience of use and non-availability at the health facilities [7]. Nurses are uniquely positioned to fill the gap by promoting this important strategy. Petkova et al. [8] reported a high level of discordance between nurses’ knowledge and their willingness to promote female condoms to their clients in Johannesburg, South Africa.

The female condom is a self-initiated sex-specific barrier method for prevention against STIs (including HIV) and unplanned pregnancy [9]. This innovative device empowers women to take full control of their protection and prevention from the double burden of STIs and unplanned pregnancy. Surprisingly, female condoms have been available and accessible to South African women since 1998; however, their impact at the population level is abysmally low [10]. Several efforts at scaling up the uptake of female condoms at the community level have proven unsuccessful in South Africa, especially in the Eastern Cape province. It initially started as a pilot project; the South African government funded the largest female condom distribution programme globally, distributing about 27 million female condoms countrywide in 2015/2016 [11].

Previous studies have documented the disproportionate use of male and female condoms in the country [11]. A South African study by Gray and Vawda (2017) [12] reported that many women were unaware of where to access female condoms. Nurses are at the forefront of healthcare and constantly engaging with women for the treatment of a myriad health conditions. They are therefore uniquely positioned to further engage with their clients about this innovative device. A previous study has shown unequivocally that adequate promotion of female condoms increases acceptability and sustained use of this barrier method among women [13].

Given the high prevalence of HIV and unplanned pregnancy in the Eastern Cape [2], female condom distribution programme may potentially prove successful in addressing these public health challenges faced in the province. However, the knowledge, attitudes and willingness of the nurses to promote female condom to clients may constitute a barrier to access at the health facility level. Thus, the critical role of nurses in creating awareness of and access to female condoms requires targeted research in the Eastern Cape province. This study sought to assess nurses’ knowledge of, attitudes to, and practices of promoting female condoms at selected primary healthcare facilities in the King Sabata Dalindyebo sub-district of the Eastern Cape. Findings from the study could inform the design of interventions on the upscaling of female condoms in the province. Also, further learning opportunities for nurses could be identified from this study.

## Methods

### Study design and setting

This descriptive cross-sectional study was conducted in April and May 2021 among nursing staff in five primary healthcare centres in the King Sabata Dalindyebo (KSD) sub-district of the Eastern Cape. Cross-sectional study offers a unique opportunity to obtain a snapshot of the study population at the specific point in time, hence, considered most suitable for this study. These health centres were purposively selected. Clients in the sub-district attend these clinics for medical care and for their contraceptive needs owing to the proximity of these clinics to many rural and urban communities. The health facilities offer a broad range of contraceptive methods such as male condoms, female condoms, intrauterine devices (IUDs), injectable contraceptives, oral contraceptives and emergency contraception. However, they do not offer sterilisation or hormonal patches.

The following community health centres (CHCs) were selected for this study: Mbekweni, Ngangelizwe, Nqanduli, Baziya, Ngcwanguba, women’s health clinic of Mthatha regional hospital, and Mthatha Gateway CHCs. Ngangelizwe clinic serves the Ngangelizwe township, the oldest township in Mthatha. Mthatha is a sprawling town with about 210 783 residents with the population level prevalence of 8–11% in the Eastern Cape [14] but many more users of its services. The CHCs offers maternity services, 24-hour casualty services for emergencies, well-baby clinics, reproductive health clinics (offering family planning and cervical screening), primary healthcare and HAST units (Centres for HIV and AIDS/sexually transmitted infections/tuberculosis).

### Study population

Overall, there were 227 nurses in the employment of Department of Health of KSD sub-district (KSD records dated 19 May, 2019). Of this total number (N = 227), 137 (60.35%) were professional nurses, 29 (12.78%) were enrolled nurses, and 61 (26.87%) were enrolled-nursing assistants. This population consisted of male and female nurses (ratio 1:5) aged between 18 and 65 years as reported in previous study [8]. This study was opened to all nurses within the study settings however, participants were excluded if they had taken scheduled leave of absence from duty (maternity, sick or vacation) during the study period.

### Sample size and technique

The sample size for this study was estimated as 143, using Cochran’s formula for a cross-sectional study.

$$N = Z1 - \alpha /22p\,(1 - p)/{d^2}$$

(where Z1 − ∝/2 is the standard normal variate at 1.96; ∝=0.05; p = 0.5).

This sample size was calculated based on the total number of nurses across the study sites (N = 227) at a confidence level of 95%, accepting a margin of error of ± 5% and a population proportion of 50%. In anticipation of missing responses or incomplete answers to questions for the key outcome measures, the estimated sample size (N = 143) was increased by 10%, bringing the total sample size to 157. A stratified random sampling technique was adopted in order to ensure inclusivity of the various categories of nurses (professional nurses, enrolled nurses and nurse assistants) and thus, eliminate recruitment bias in the study.

### Study procedure

For inclusiveness, all the nurses were stratified by health facility and professional category (professional nurses, enrolled nurses and nurse assistants). The nurses’ register was obtained at the various health facilities and names were stratified into the various categories. A random sample of the nurses corresponding to the sizes of the strata was recruited by using a drawing lots. Participants were randomly selected within the various professional categories in each facility.

Prior to data collection, a scheduled visit to each CHC was undertaken after telephonic agreement on the date with the facility manager. The selected participants were provided with an information sheet outlining the purpose, procedure, potential risks, and benefits of the study, as well as their rights to withdraw at any time, which were clearly explained to them.

Quantitative data were obtained by using a structured pre-tested self-administered questionnaire written in English. The questionnaire was developed based on the objectives of the study and comprised three parts; sociodemographic factors, knowledge about female condoms, and attitude to the promotion of female condoms during patient encounters. The questionnaire was piloted with five nurses in a similar setting to the study setting. The barriers to promoting female condoms to their clients were obtained qualitatively in the pilot test and the responses were later re-coded and categorised into six themes. These themes were converted to respondent-options (quantitative data) in the questionnaire for the main study (Suppl. 1). The final instrument was assessed by the authors (EE, PY & OVA) for face and content validity.

## Measures

### Outcome measures

Knowledge and attitudes of nurses about female condoms were assessed by nurses’ self-reporting in the questionnaire, adapted from Petkova et al. [8]. The questions assessed the knowledge of important issues about female condoms, with respondents answering ‘True’ or ‘False’ to each item. For instance, participants responded to ‘Female condoms do not prevent pregnancy and STIs’, ‘Female condoms cannot be used simultaneously with male condoms’, and others. Another set of questions focused on the counselling tips offered by nurses to their clients. For instance, ‘The client should not feel the female condom in her body’ and ‘Provide counselling in either individual or small group sessions with other women providing peer support’. Respondents answered ‘True’ or ‘False’ to each statement. Participants were subsequently categorised as knowledgeable if they answered at least three of the five knowledge questions correctly. The nurses’ attitudes were assessed through their answers on the questionnaire. They self-assessed themselves as ‘more positive or less positive’ about female condoms, and each participant self-reported the perceived barriers to promoting female condoms to their clients.

### Covariates

Relevant items on sociodemographic characteristics were sex, age, marital status, religious affiliation, professional categories, and years of nursing experience. Age and years of nursing experience were categorised in decades, while professional categories were classified as professional nurses, enrolled nurses and enrolled nursing assistants. Participants were asked about their sources of information on female condoms and the form of contraception they most often recommended to their clients.

### Quantitative data analysis

Data were analysed with the IBM Statistical Package for Social Sciences Version 27.0 for Windows (IBM Corp., Armonk, NY, USA). Only complete responses were included in the final analysis (Suppl. 2). Frequency counts and percentages were estimated for categorical variables and means (standard deviation) were estimated for continuous variables. Given the binary nature of the main outcome measure (Yes or No response), the Pearson chi-square test was used to assess the associations between the nurses’ knowledge, attitudes and practice/willingness to promote the female condom to their clients. A *p*-value of less than 0.05 was considered statistically significant.

## Results

### Baseline characteristics

Of the 157 nurses selected for the study, a total of 139 across five health facilities gave complete responses (a response rate of 88.5%). The majority of the participants were females (76.3%), within the age range of 30–59 years (75.6%) and single (59.0%). Most of the participants were professional nurses (60.4%), followed by enrolled nursing assistants (22.3%) and enrolled nurses (17.3%). Nearly all the participants were Christians (98.6%). The majority of the participants had fewer than ten years’ professional experience (69.8%), while 10 participants had over 20 years’ professional experience (Table 1).

**Table 1:** Baseline characteristics of the participants

Demographic variables	Frequency (N = 139)	Percentage (%)
**Sex**		
Male	33	23.7
Female	106	76.3
**Age distribution**		
Less than 29 years	31	22.3
30–39 years	36	25.9
40–49 years	50	36.0
50–59 years	19	13.7
60–69 years	3	2.2
**Marital status**		
Single	82	59.0
Married	47	33.8
Cohabiting	2	1.4
Divorced	1	0.7
Widow	7	5.0
**Religious affiliation**		
Christian	137	98.6
No religious affiliation	1	0.7
African religion	1	0.7
**Professional status**		
Professional nurse	84	60.4
Enrolled nurse	24	17.3
Enrolled nursing assistant	31	22.3
**Years working**		
Less than 10 years	97	69.8
10–19 years	32	23.0
20–29 years	8	5.8
30 + years	2	1.4

Source: created by the authors

### Awareness of the female condom among nurses

**Fig. 1:**
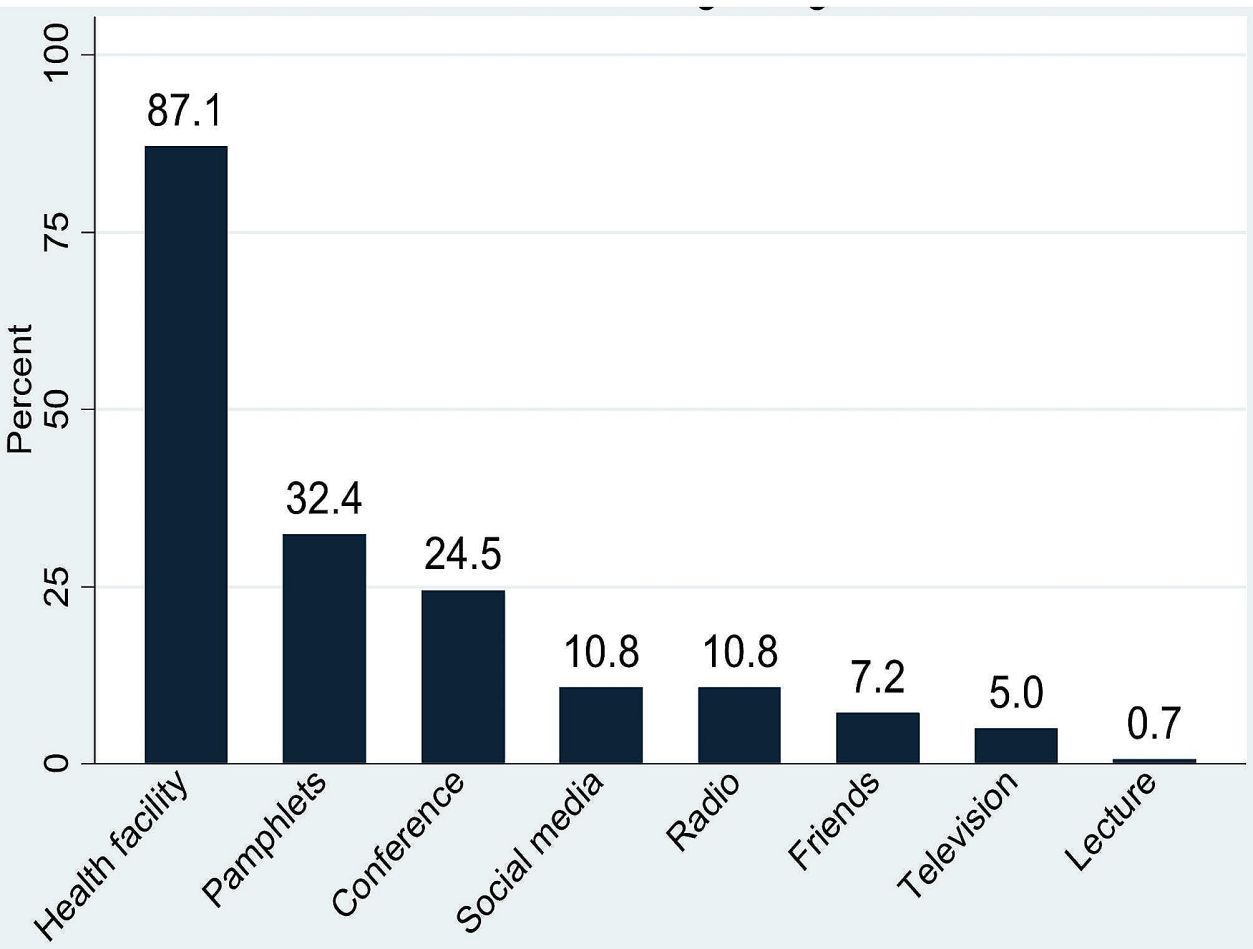
Main sources of information on the female condom

All the participants (N = 139) reported that they had heard of female condoms, and 83.5% of them had seen a female condom outside its wrapping (n = 116). The source of information about the female condom was predominantly the health facility (87.1%), while pamphlets (32.1%) and conferences (24.5%) were additional sources of information on female condom use (Fig. 1).

### Contraceptive methods recommended by the nurses

**Fig. 2:**
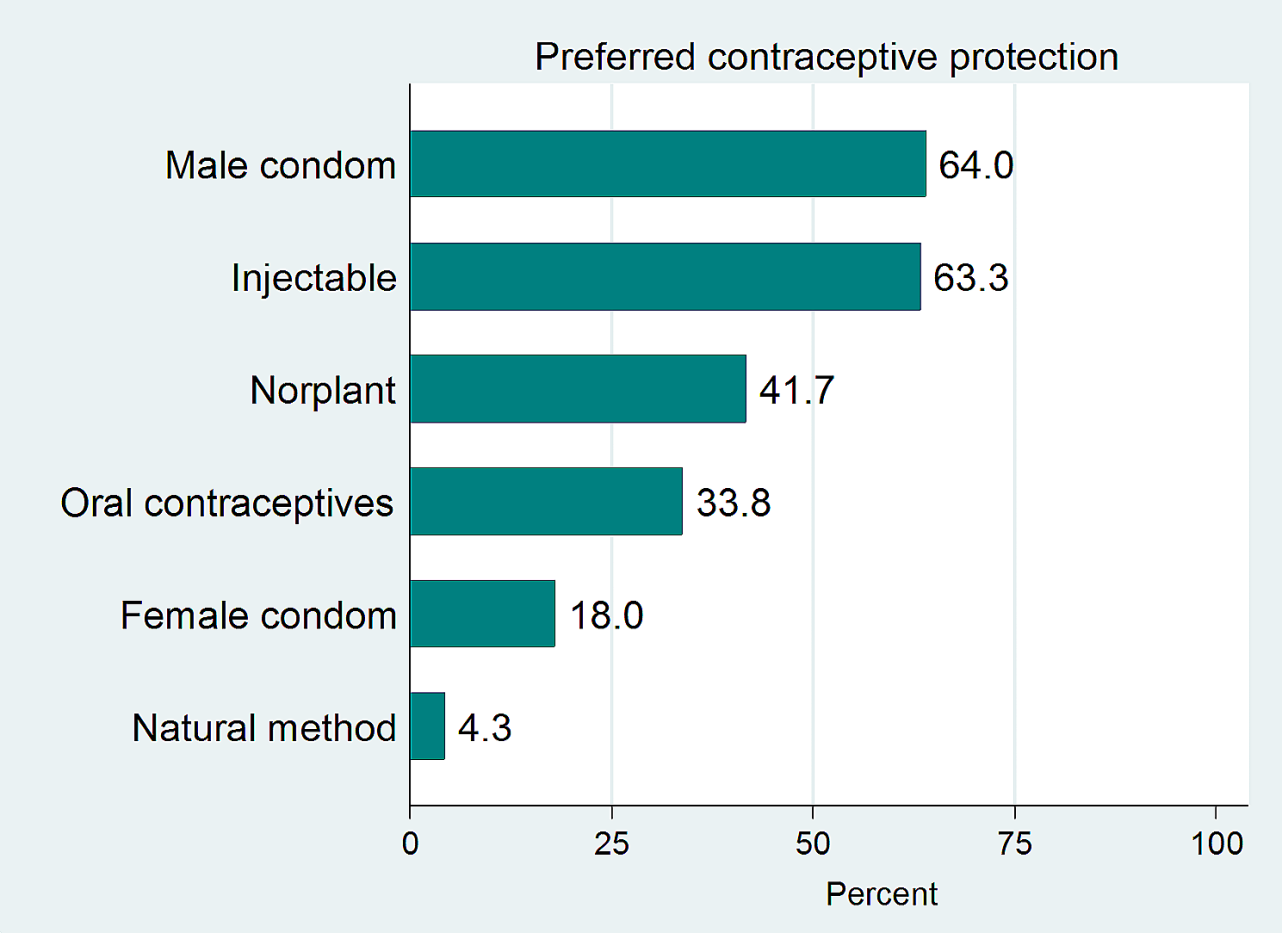
Contraceptive methods recommended by the participants

The majority of the participants preferred to recommend the male condom (64%) and hormonal injectables (63%) to their clients, while the female condom (18%) and the natural method (4.3%) were the least recommended methods (Fig. 2).

### Knowledge of and counselling tips on the female condom

Nearly all the participants (95.7%) knew that the female condom (FC) prevents pregnancy and STIs. In total, 63.8% of the participants agreed that female condoms could not be used with male condoms. However, fewer nurses knew that the FC does not reduce sexual pleasure (14.4%) and can be inserted eight hours prior to intercourse (28,3%). Very few nurses understood the correct counselling advice on whether women should feel the condom in their genitals (18.1%) and a similarly low number reviewed the anatomy and physiology of the body with their clients (27.5%). However, a large proportion of the nurses recognised the need to give individual counselling or small group counselling (96.4%), understood that the FC is slippery to work with (95%) and would typically need to be used two to three times before a woman is comfortable with its use (79.1%) (Table 2).

**Table 2:** Nurses’ knowledge and counselling tips on the use of the female condom

Variables of interest	True n (%)	False n (%)
FC does not prevent pregnancy and STIs (n = 138)	6 (4.3)	132 (95.7)
FC cannot be used simultaneously with the male condom (n = 138)	88 (63.8)	50 (36.2)
FC cannot be inserted eight hours before intercourse (n = 138)	39 (28.3)	99 (71.7)
FC removes sexual arousal (n = 138)	20 (14.4)	118 (85.6)
The FC has a flexible ring at the closed end of the pouch with a slightly larger ring at the open end (n = 138)	121 (87.7)	17 (12.3)
**Counselling tips**	**TRUE n (%)**	**FALSE n (%)**
The client should not feel the FC in her body (n = 138)	25 (18.1)	113 (81.9)
It is not necessary to review the female anatomy and physiology (n = 138)	38 (27.5%)	100 (72.1)
Provide counselling in either individual or small group sessions with other women providing peer support (n = 138)	134 (96.4)	5 (3.6)
Provide insertion, demonstration, and practice on models (n = 138)	133 (96.4)	5 (3.6)
Counsel the client that FC may be slippery to work with at first but becomes easier with practice (n = 138)	131 (95.0)	7 (5.0)
It can take 2–3 times to be fully comfortable using FC (n = 138)	110 (79.1)	28 (20.9)

Source: created by the authors

### Association of demographic characteristics and knowledge of the FC

The majority of the nurses (82.7%) were knowledgeable about the female condom. The Ngangelizwe and Mthatha Gateway clinics’ participants were significantly (p = 0.002) more knowledgeable about female condoms than participants from the other facilities. The majority of the participants (84.2%) answered two of the knowledge questions correctly: ‘Female condoms do not prevent pregnancy and STIs’ and ‘The female condom removes sexual arousal’. There was a statistically significant difference (p = 0.003) between the knowledge levels of the various professional categories; the highest proportion of individuals who answered the knowledge questions correctly (and were deemed ‘knowledgeable’) were professional nurses (Suppl. 3).

### The attitudes of nurses towards the use of the female condom

The participants’ attitudes were positive with regard to the female condom’s ability to protect against unwanted pregnancies and its relative ease of use. Also, they had positive views with regard to the female condom’s ability to empower women by giving them control over their sexual decisions and bodies. The Pearson’s chi squared test showed that participants working at Ngangelizwe healthcare centre were more likely than those working at the Baziya clinic to have a positive attitude towards the female condom (Table 3).

### Association between the level of knowledge and willingness to promote the FC

Despite 83.3% of the participants being knowledgeable about the FC, a negative association was found between level of knowledge and willingness to promote the FC. About half of the participants reported that they did not promote the FC among their clients (50.7%). Participants who were more knowledgeable were less likely to promote the FC to their clients than those who were less knowledgeable (p = 0,012). Again, a high proportion of the participants were knowledgeable about the prophylactic and sexual enhancement of the FC (84.7%), but there was no significant corresponding willingness to promote the FC to their clients. Also, a high proportion of the participants had positive attitudes towards the use of the FC, but this did not make any difference to their willingness to promote it at their clinics. The majority of the participants (64.5%) reported that they did not have a judgemental attitude towards the use of the FC, but there was no significant willingness to promote the FC at their facility (Table 3).

**Table 3:** Association between the knowledge of the FC and willingness to promote the use of the FC

Variable of interest	Willingness to promote (n = 68)	Not willing to promote (n = 70)	*p*-value
Knowledge level			
More knowledgeable n = 115	51 (44.3)	64 (55.7)	0.012
Less knowledgeable N = 24	18 (75.0)	6 (25.0)	
Knowledge of prophylactic and sexual enhancement uses			0.463
Knowledgeable n = 117	56 (47.9)	61 (52.1)	
Not knowledgeable n = 22	13 (59.1)	9 (40.9)	
Attitude level			0.442
More positive n = 108	56 (51.9)	52 (48.1)	
Less positive n = 31	13 (41.9)	18 (58.1)	
Healthcare workers have no judgmental attitude			0.584
Agree n = 89	47 (52.8)	42 (47.2)	
Disagree n = 33	15 (45.5)	18 (54.5)	
Unsure n = 17	7 (41.2)	10 (58.8)	

Source: created by the authors

### Barriers to promoting female condoms

Most of the participants (76.3%) believed that poor knowledge about female condoms was the main barrier to its promotion. Other reasons advanced by the participants for not promoting the FC to their clients included lack of availability (41.0%), low adoption by the clinic’s clients (16.5%) and others (Suppl. 4).

## Discussion

Given the unmet need for contraception that offers dual protection against unplanned pregnancies, HIV and other STIs in South Africa, especially in the rural Eastern Cape, female condom (FC) use should be promoted in the province. Nurses serve in the frontline of healthcare and are uniquely positioned to promote this innovative strategy to their clients in the various primary healthcare centres in the country. This study therefore examined nurses’ knowledge of and attitudes to the use of the FC, and their willingness to promote the FC to their clients in selected primary healthcare centres in the KSD sub-district of Eastern Cape Province, South Africa.

Overall, there was a high level of awareness of the FC among nurses; all the participants had heard of the female condom, and a great majority (83.5%) had seen it outside its wrapping. This is not surprising given the huge investment of the government in making the FC available in all the PHCs in the country [12]. Just over half of the nurses had been exposed to the FC during their training (51%), although most (87.1%) confirmed that PHCs were their main source of information about the FC. In addition, more than half of the nurses (57%) had gained experience in the family planning unit of the PHC in which they worked. Also, about half of the participants had received formal training on the use of the FC, and skills transfer within the family planning unit was reported by 61% of the participants. These findings corroborate a previous studies [15, 16] which showed that healthcare providers play a cardinal role in influencing the initiation and continued use of the FC. However, issue of shortage of nurses at the PHCs requires attention of health authorities in order to scale up the use of FC in the country. Evidence suggests that shortages of nurses impact service delivery, including creating awareness, counselling and promoting the use of FC at the PHCs [17]. In order to further strengthen access to the FC in the region, awareness campaigns through electronic and print media should be combined with direct engagement via conferences and workshops to target nurses and the general population.

Interestingly, this study found that the majority of the participants were knowledgeable about FC across all the knowledge domains. The majority (95.7%) knew that female condoms prevent pregnancy and STIs. This finding corroborates previous reports, [18] although the figure is slightly higher (95.7% versus 88%). Sixty-three per cent of the participants knew that the female condom should not be used simultaneously with the male condom. This result is better than that of a previous study [18] among a similar population in Gauteng, South Africa, which showed that more than half of the participants answered the question incorrectly. Regarding whether the FC could be inserted up to eight hours before intercourse, 71.7% of the participants correctly answered this question. This is similar to the finding of Petkova et al., [18] who reported that 74% of the participants correctly responded to this question. Eighty-five per cent of participants correctly answered that the female condom does not remove sexual arousal, which is also similar to Petkova et al., [18] who reported that 91.26% of respondents correctly answered this question. Regarding the structure of the FC, the majority of the participants (87.7%) correctly answered this question, which is similar to 88% reported by Petkova et al. [18] However, a significant difference exists between levels of knowledge in the various professional categories; enrolled nursing assistants and enrolled nurses had lower levels of knowledge than professional nurses. Targeted specialised training on the FC for junior nurses could bridge the knowledge gaps identified in this cohort. In addition, nurses should be equipped with more information on the effect of FC on sexual arousal, timing of insertion of FC prior to sex, and female anatomy.

Despite the nurses’ overall agreement that the FC offers protection against unplanned pregnancies and empowers women to take control of their bodies and their sexual decisions, a negative attitude was observed with regard to promoting the use of the FC. This is reflected largely in their choices of which contraceptive methods to recommend to their clients. Most recommended the male condom, the injectable or Norplant ahead of female condoms. Perhaps, a qualitative study component could have elucidated more on the underlying reasons for their preferences. Oliveira et al. [19] noted that health professionals believed that women do not know where to obtain FC nor use it. More so that male condom is much more used and easier to get than FC. Bekinska et al. [20] reported that willingness to promote the FC can lead to an increase in the uptake of this strategy. While most participants were of the view that healthcare workers do not hold judgmental attitudes towards FC users, some noted that poor knowledge of the FC is a barrier to its promotion. Interestingly, a more positive attitude towards the FC was found among nurses at Ngangelizwe healthcare centre than in the other clinics. It is unclear if the attitudes of the nurses who withdrew (34.8%) differ from those who participated in this facility. Nonetheless, more studies are needed to elucidate the variations in the attitude of the nurses from one facility to another in the region.

These findings show the importance of positive attitudes among nurses when it comes to promoting the use of the FC. Knowledge alone is clearly not enough to motivate nurses to promote its use. Poor attitudes towards the FC have also been documented by Peters et al. [13] who established that there is lack of acceptance of the female condom by healthcare workers in several countries such as South Africa, Kenya and the USA. Chipfuwa et al. [21] and Weeks et al. [22] highlight nurses’ minimal efforts and negative attitudes with regard to promoting and raising awareness about the female condom in Zimbabwe and the USA, respectively.

Thus, it is clear that high levels of awareness of the FC among the nurses did not translate into willingness to promote this strategy to their clients. Some participants believed that poor knowledge about FC was a barrier to promoting the device to their clients. Mantell et al. [15] emphasised the pivotal role healthcare providers play in disseminating knowledge and increasing the use of the FC for HIV and STI prevention. Ananga et al. [23] also highlighted the role of nurses in spreading FC education and promoting its use. In order to harness their role and ensure successful expansion of this service, nurses need both improved knowledge of the benefits of the FC and improved attitudes towards promoting it. Among other barriers, non-availability (41.0%) is one of the reasons for not promoting FC at the facilities. However, non-availability is inherently linked to failure to request the FC for distribution at the health facilities. All the health facilities receive stock of medications and other consumables from the Eastern Cape provincial depot. Therefore, facility managers must monitor stocks of FC and ensure availability through their weekly and monthly audit of orders.

It is not altogether clear why those with good knowledge of the FC were unwilling to promote its use. A plausible explanations could be that nurses are overburdened by long queues at clients and have too little staff to provide a wide range of services at the various PHCs. They may lack the energy required to promote a concept that has not yet attained widespread acceptance, and so choose to go with what people already know. The findings from this study are similar to those of a South African study [24] which found that although healthcare workers play an important role in promoting reproductive health services, staff shortages and long patient queues make it difficult for communities to benefit from reproductive health information. Using the case of Botswana, Mashanda-Tafaune and Monareng [25] posited that high patient loads and shortages of staff resulted in poor promotion of reproductive health services, including female condoms.

### Strengths and limitations

This study highlighted important issues for interventions in order to expand the use of FC in the region and country. Awareness campaigns through electronic and print media should be combined with direct engagement with nurses and the general population. These strategies have been used successfully to improve FC uptake in Tanzania [26] and Zimbabwe [27]. Given that the majority (61.2%) of the total nursing workforce in the sub-district participated in this study, the findings reflect the overall knowledge, attitudes and willingness of nurses to promote FC in the region. However, the limitations of the study cannot be ignored. It is unclear if some or all of the nurses who withdrew from the study had negative attitudes toward FC. A mixed-methods design would have allowed qualitative exploration of the reasons for the poor attitudes and low levels of FC promotion. Future studies should explore the perspective of the broader population of women in both rural and urban communities in the Eastern Cape on the use of the FC.

## Conclusions

This study found good knowledge of the female condom among the nurses; however, the knowledge does not show in their willingness to promote this device to their clients. Knowledge gaps on the female condom exist among the junior nurses. In addition, health facilities were identified as the main source of information on female condom. Targeted training according to the professional category of the nurses will be crucial for capacity building among nurses in the region. Awareness campaigns on the use of the female condom through electronic and print media should be combined with direct engagement of the nursing staff in order to change their attitudes to the promote of the FC to their clients. Further studies should evaluate the content of training programmes on the female condom in order to identify gaps and propose feasible solutions.

### Electronic supplementary material

Below is the link to the electronic supplementary material.

Supplementary Material 1

## Data Availability

The data supporting the findings of this study are available from the corresponding author (EE) on reasonable request.
