# Supplementary material for: Assessing nurses’ knowledge and attitudes towards promoting female condom use in South African primary healthcare clinics
Source: BMC Health Serv Res. 2024 Jan 5;24:35. doi: 10.1186/s12913-023-10504-9 (PMC10770987; doi:10.1186/s12913-023-10504-9)
Supplement: Supplementary file 1 — Supplementary Material 1 [file 12913_2023_10504_MOESM1_ESM.docx]

**Supplementary**

Suppl. 1: Study Questionnaire


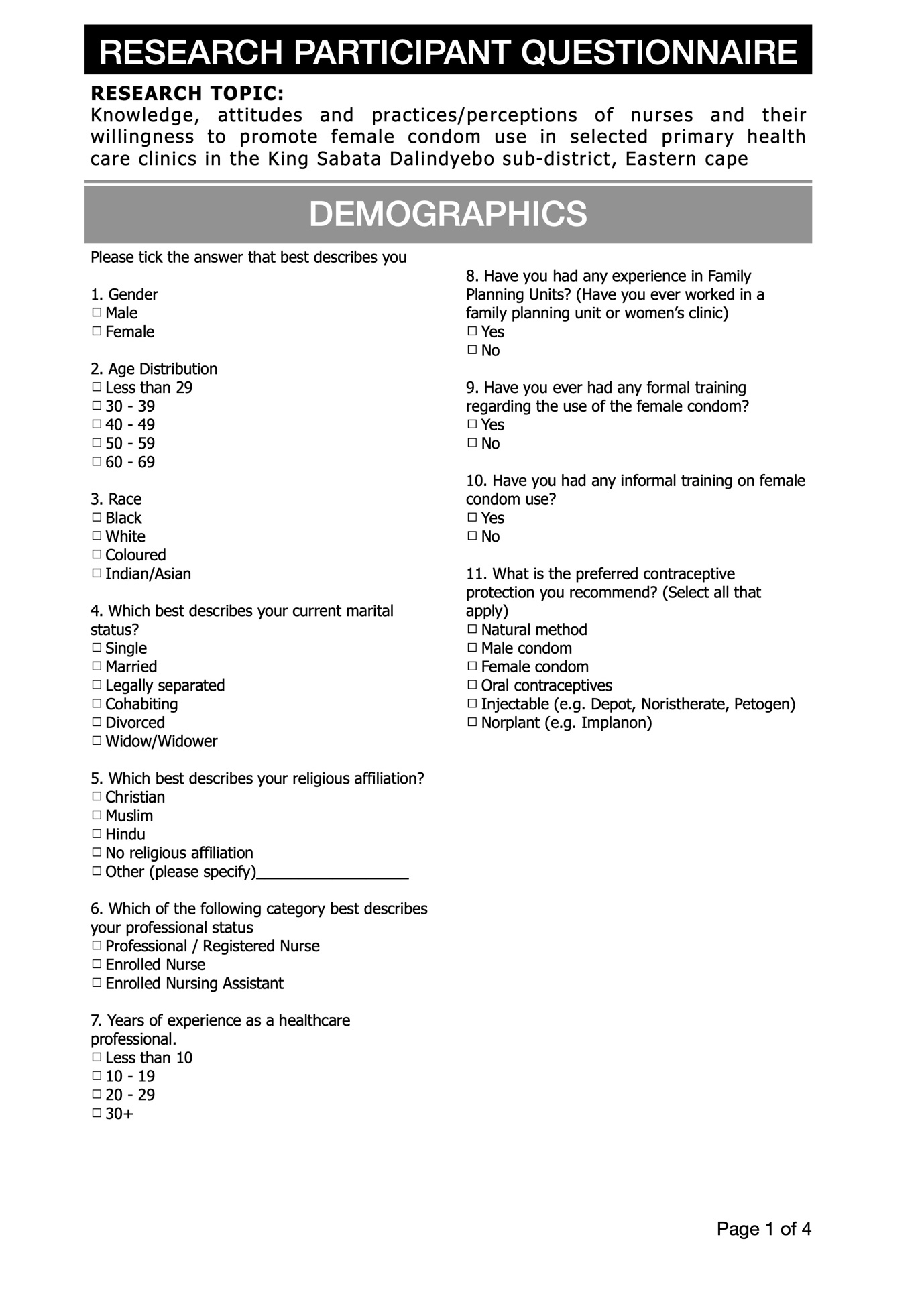


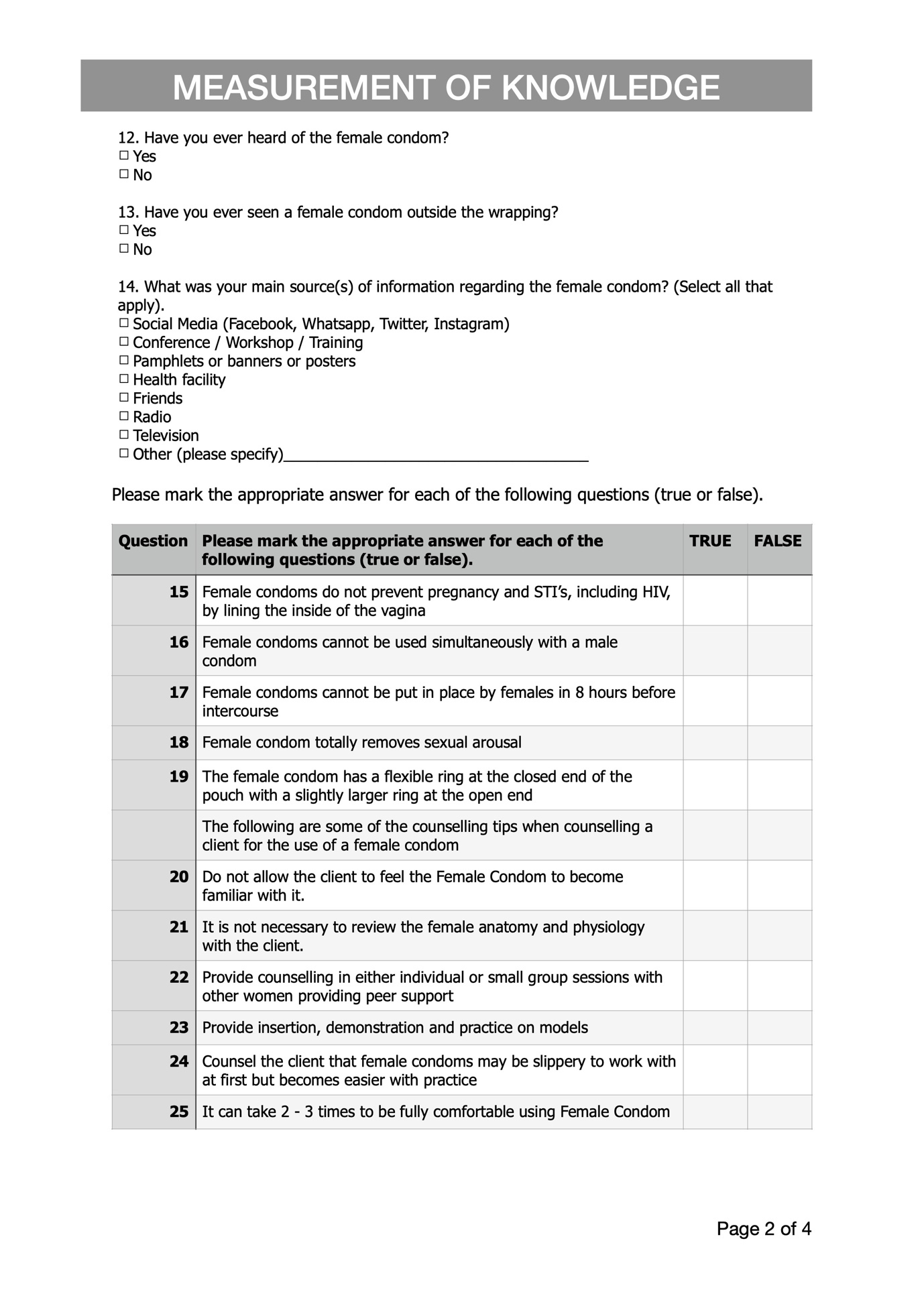


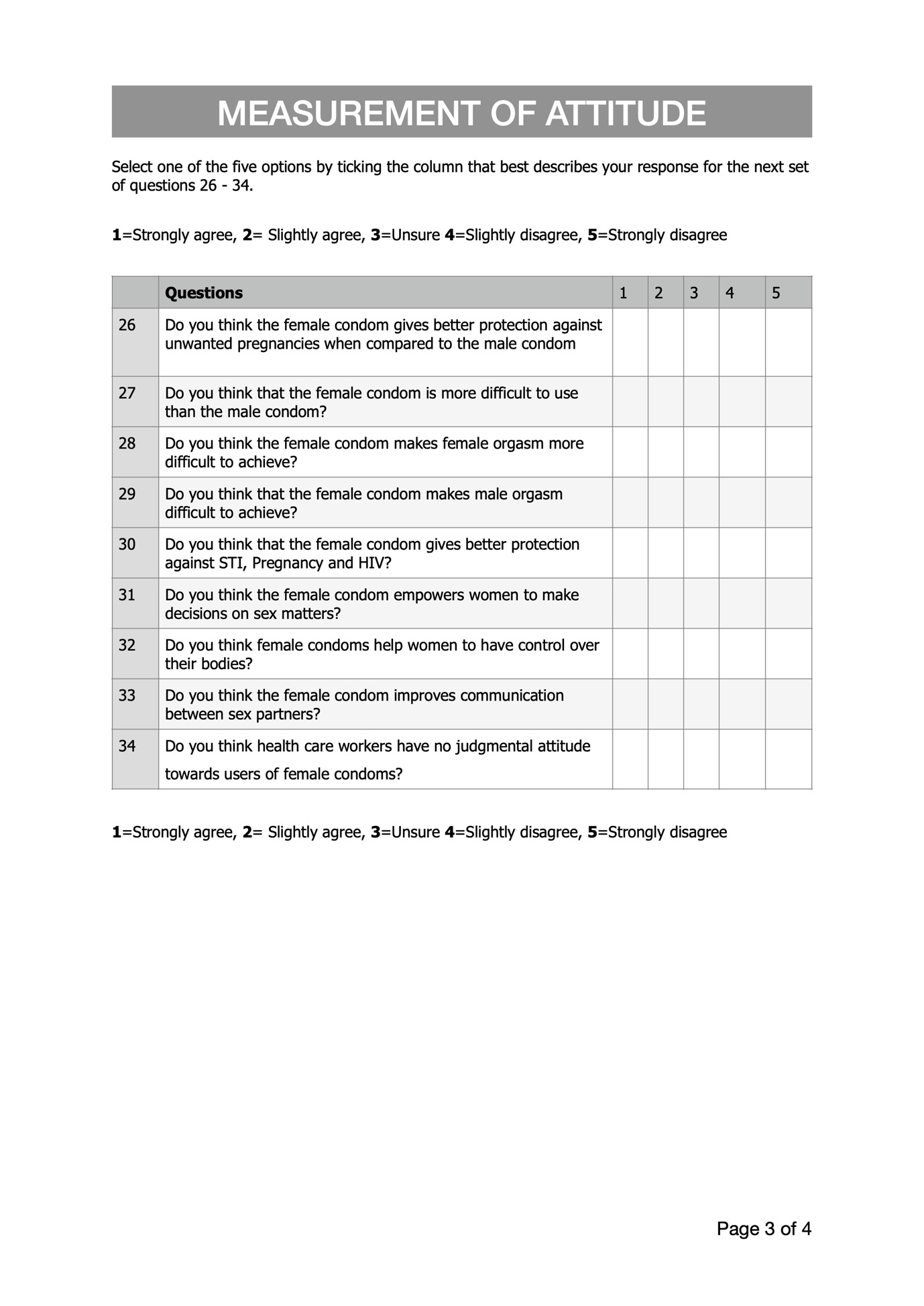


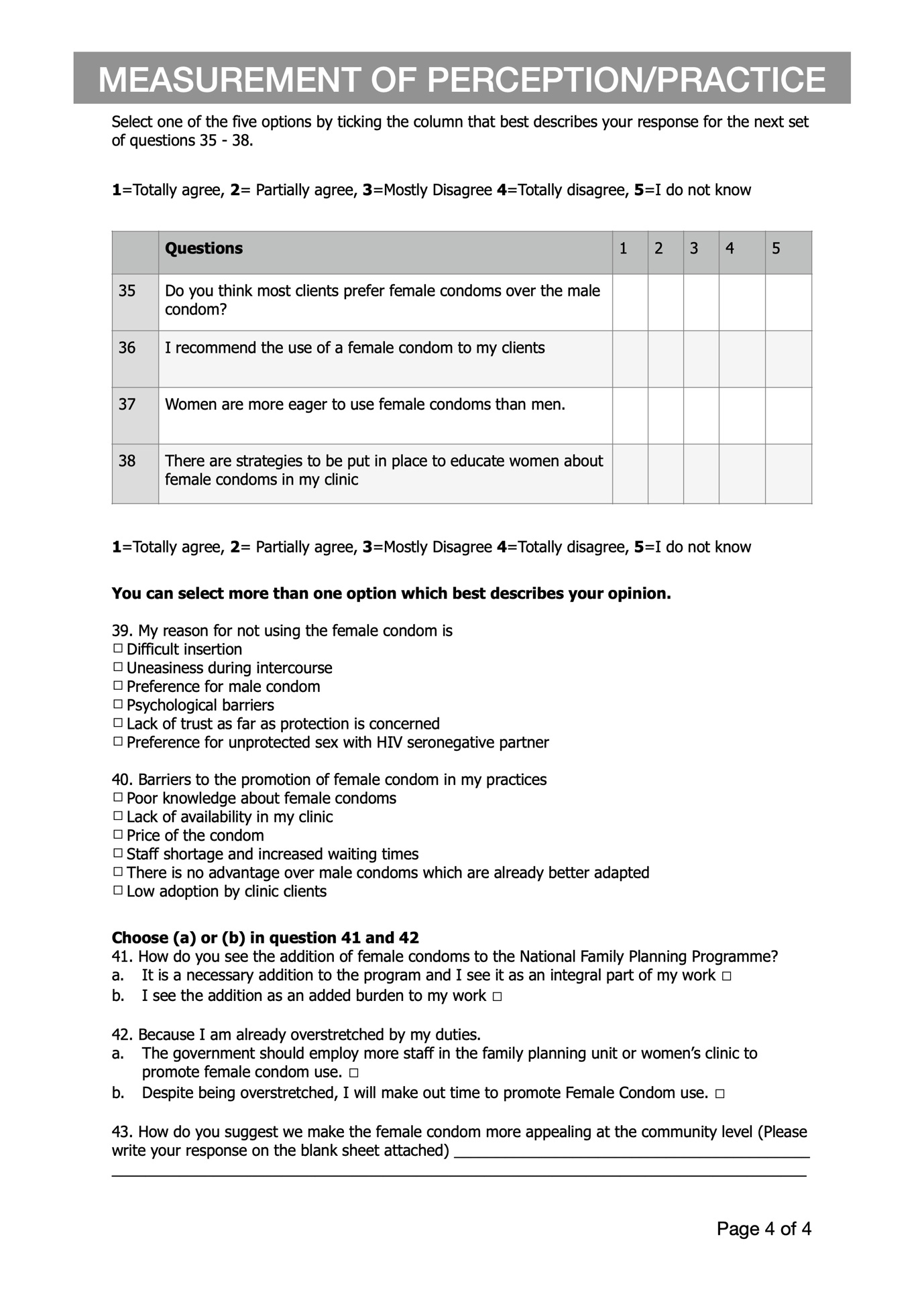


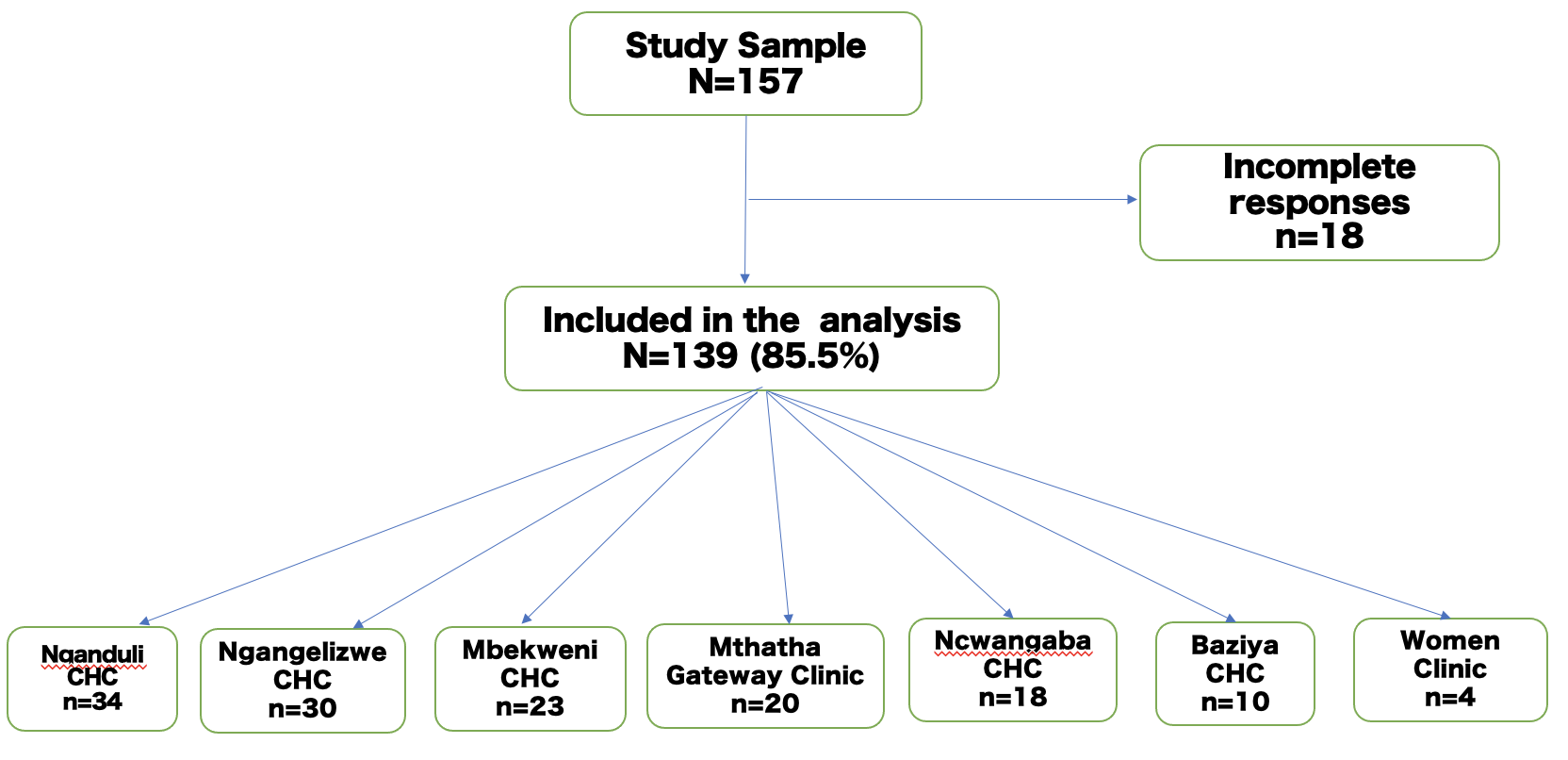


Suppl. 2: Flow diagram of recruitment process

Suppl. 3: Knowledge by professional category (p=0.003)

Suppl. 4: Barriers to the promotion of female condoms in healthcare practices
